# P-293. Epidemiology of Extraintestinal Invasive *Escherichia coli* Infections in 9 U.S. Communities, 2023

**DOI:** 10.1093/ofid/ofae631.496

**Published:** 2025-01-29

**Authors:** Heather N Grome, Joshua M Brandenburg, Julian E Grass, Erin Parker, Helen Johnston, Jennifer Driscoll, Paulina Rebolledo, Gillian Smith, Lucy E Wilson, Emily Luckman, Jennifer Zipprich, Marco Garcia, Marisa Hoffman, Kristina Flores, Hsioa Che Looi, Julia Tellerman, Shannon C O’Brien, Daniel Muleta, Olivia Denzie, Alice Guh

**Affiliations:** Centers for Disease Control and Prevention, Atlanta, Georgia; Centers for Disease Control and Prevention, Atlanta, Georgia; CDC, Atlanta, GA; California Emerging Infections Program, Oakland, California; Colorado Department of Public Health, Denver, Colorado; Colorado Department of Public Health and Environment, Denver, CO, Denver, Colorado; Emory University School of Medicine, Emory University Rollins School of Public Health, Atlanta, GA; Georgia Emerging Infections Program, Atlanta, GA; Foundation for Atlanta Veterans Education and Research, Decatur, GA; Atlanta Veterans Affairs Medical Center, Decatur, GA, Atlanta, Georgia; University of Maryland Baltimore County, Baltimore, Maryland; Maryland Department of Health, Baltimore, Maryland; Minnesota Department of Health, St. Paul, Minnesota; Minnesota Department of Health, St. Paul, Minnesota; University of New Mexico, Albuquerque, New Mexico; University of New Mexico, New Mexico Emerging Infections Program, Albuquerque, New Mexico; New York Rochester Emerging Infections Program at the University of Rochester Medical Center, Rochester, New York, Rochester, New York; New York Emerging Infections Program, Rochester, New York; Oregon Health Authority, Portland, Oregon; Tennessee Department of Health, Nashville TN, Antioch, Tennessee; Tennessee Department of Health, Nashville, Tennessee; Centers for Disease Control and Prevention, Atlanta, Georgia

## Abstract

**Background:**

Extraintestinal invasive *Escherichia coli* (iEC) is a leading cause of sepsis and hospitalization, but US surveillance for iEC has been frequently limited to multidrug-resistant (MDR) strains and hospitalized cohorts. To describe the incidence and clinical characteristics of MDR and non-MDR iEC and inform prevention and vaccine development, CDC’s Emerging Infections Program piloted active population- and laboratory-based surveillance in 9 US sites.

**Methods:**

Among surveillance area residents ( > 7.3 million people), an incident iEC case was the first isolation of *E. coli* in a 30-day period from a normally sterile body site (June–August 2023). Demographic, clinical, and laboratory characteristics were assessed by chart review. Annual incidence rates by surveillance area were estimated by multiplying total case number by 4 and used 2022 US census data for denominators.

**Results:**

Among 1345 iEC cases in 1334 patients, *E. coli* was isolated from blood in 1223 (90.9%) and from other sterile sites in 122 (9.1%). Median age was 68 years (IQR 55–79); 766 (57.0%) were female. Overall estimated annual crude incidence rate was 74.5 cases per 100,000 population (range by area 51.3–95.6) and was higher for persons aged ≥ 60 vs < 60 years (228.5 vs 30.4). Cases were most commonly community associated (554, 41.2%) or health care associated community onset (633, 47.1%). Most case-patients (1194, 88.8%) had comorbidities; diabetes was most common (457, 34.0%). Of all cases, 762 (56.7%) were associated with urinary tract infection (UTI), 192 (14.3%) with recurrent UTI, and 213 (15.8%) had a urinary catheter in the 2 days before collection of the iEC-defining specimen. Among 1160 hospitalized cases, median length of stay was 5 days (IQR 3–9), 103 (8.9%) died. Of reported *E. coli* susceptibilities, 144/500 (28.8%) were fluoroquinolone-resistant, 185/1345 (13.8%) were extended-spectrum β-lactamase producing, and 3/1345 (0.2%) carbapenem-resistant.

**Conclusion:**

The burden of iEC disease was substantial compared to other invasive pathogens and primarily affected older adults. Over half of infections accompanied UTIs and involved hospitalization. Continued surveillance to monitor trends over time, inform vaccine development and evaluation, and advise prevention efforts are needed.

**Disclosures:**

**All Authors**: No reported disclosures

